# Supplementary material for: Inactivation of NF-κB2 (p52) restrains hepatic glucagon response via preserving PDE4B induction
Source: Nat Commun. 2019 Sep 20;10:4303. doi: 10.1038/s41467-019-12351-x (PMC6754499; doi:10.1038/s41467-019-12351-x)
Supplement: Supplementary file 1 — Supplementary Information [file 41467_2019_12351_MOESM1_ESM.pdf]

Supplementary Information

Inactivation of NF-κB2 (p52) restrains hepatic glucagon response via preserving PDE4B induction

Zhang et al.

Supplementary Materials and Methods

Oral glucose tolerance test

After an overnight fast, HFD-fed mice were given glucose (2 g/kg body weight) by gavage. Blood glucose levels were measured using ONE-TOUCH glucose meters (Johnson, America) from tail tips at indicated times.

Liver-specific knockdown of p52

p52 liver-specific knockdown mice were created using AAV8-shRNA technology. In brief, 8-week-old C57BL/6J mice were injected with 7 μL AAV8-shRNA solution (virus titer > 10^13) diluted with 193 μL normal saline through tail vein. Mice in control groups were injected with AAV8-Negative Control shRNA (AAV8-NC shRNA). 3 weeks later, mice were randomly divided into different groups for glucagon challenge experiments.

The shRNA oligo we used in our experiments are as follows:

Negative control shRNA: TTCTCCGAACGTGTCACGT

p52 shRNA: GCAGATAGCCCACGTCATTTA

Short-term HFD-fed

6-8-week-old male C57BL/6J mice were purchased from Sino-British SIPPR/BK Lab. Animal Ltd (Shanghai, China). After one week of acclimation, mice were knocked down p52 using siRNA transfection *in vivo* technology as described. 48h after the injection, mice were fed with a high-fat diet (HFD, 60% kcal from fat; D12492; Research Diet, America) or a normal chow diet (NCD, 10% kcal from fat; Xietong Organism, China) for 5 days. Food intake was recorded every day. Pyruvate tolerance test was performed on overnight fasting mice as previously described. The procedures

for experiments and animal care were approved by the Institutional Animal Care and Use Committee of China Pharmaceutical University (Nanjing, China).

Quantitative real-time reverse transcription PCR (qRT-PCR)

Total mRNA was isolated using TRIzol™ Reagent (Invitrogen, America) from the livers of mice, primary hepatocytes or HepG2 cells and cDNAs were synthesized. qRT-PCR was performed on the Roche LightCycler 96 System using the Fast SYBR Green Master Mix (Roche, America). The mRNA expression levels of target genes, including *NF-κB2*, *G6Pase*, *PEPCK*, *PGC-1α*, *SREBP-1*, *HMGCR*, *ABCA5*, *FASN*, *ACC*, *HSL*, *Acsl1*, *Lipc*, *Acadl* and *PNPL2* were normalized to *β-actin* expression levels. All the primer pairs used are listed in Supplementary Table 1.

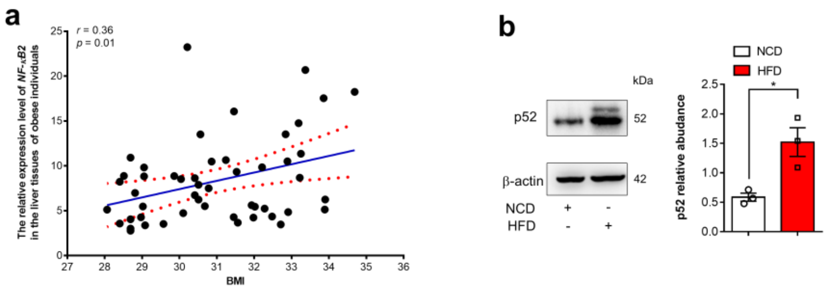

Supplementary Fig. 1 Obesity activates *NF-κB2* expression in human liver samples. (**a**) *NF-κB2* expression was significantly related to the BMI in the 51 liver samples from obese individuals using the Genotype and Phenotypes (dbGaP, phs000424.v7.p2) database. The Pearson correlation coefficient between the relative expression level of NF-κB2 in the liver tissue of obesity individuals and BMI was calculated using GraphPad Prism (version 6.02). (**b**) Western blotting analysis of p52 expression in liver samples from mice fed with HFD, β-actin levels used as control for normalization. Bars represent mean ± SEM values. BMI: body mass index; NCD: normal chow diet; HFD: high fat diet. Statistical differences between pairs of groups were determined by a two-tailed Student's *t*-test. *: $p < 0.05$ *vs.* the control group. Source data are provided as a Source Data file.

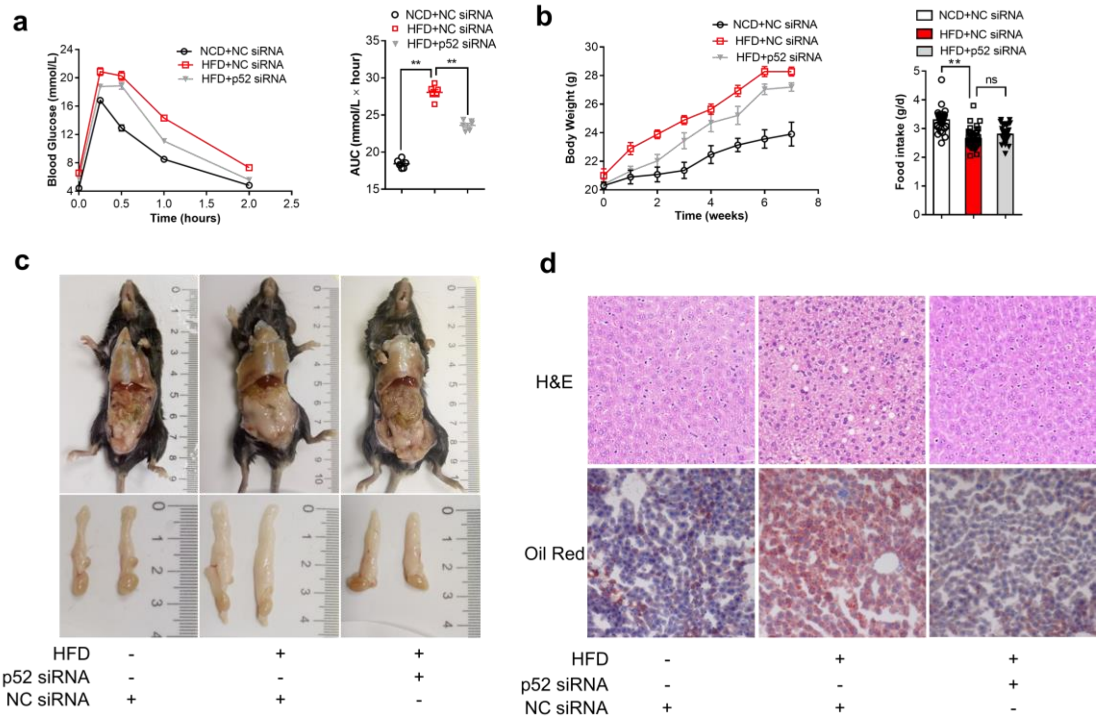

Supplementary Fig. 2 p52 siRNA alleviates lipid accumulation in livers of HFD-fed mice. (**a**) Oral glucose tolerance test (2 g/kg body weight) after 7 weeks of feeding (n=6). (**b**) Body weight and food intake of negative control or p52 siRNA injected mice fed with NCD or HFD. (**c**) Epididymal fat pad before (upper panel) and after (lower panel) dissection. (**d**) H&E and oil red O staining in liver tissue (original magnification, ×40). Bars represent mean ± SEM values. NCD: normal chow diet; HFD: high fat diet; H&E: hematoxylin and eosin. Bars represent mean ± SEM values. Statistical differences were determined by one-way ANOVA. *: $p < 0.05$, **: $p < 0.01$ *vs.* the control group. ns: not statistically significant. Source data are provided as a Source Data file.

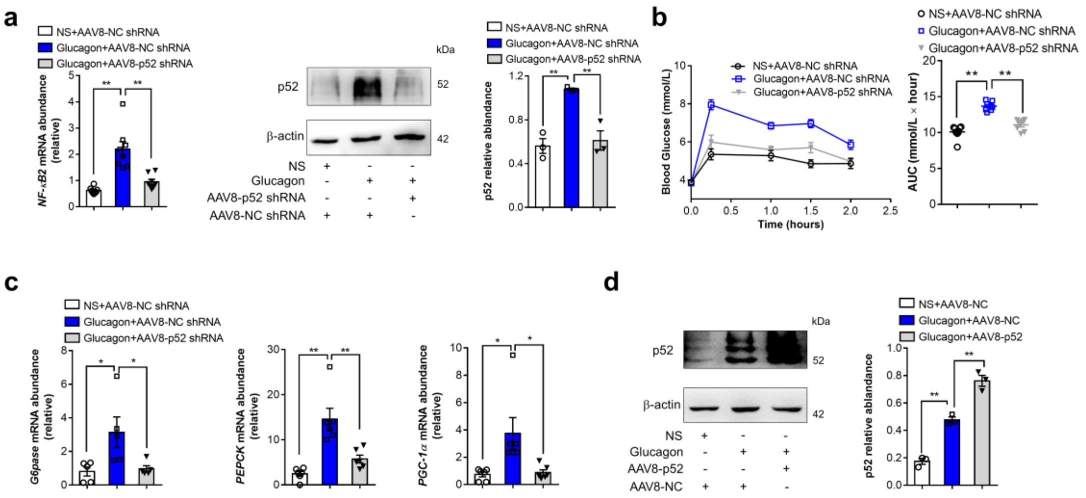

Supplementary Fig. 3 Liver-specific silencing of p52 suppresses hepatic glucagon response in mice. (**a**) Relative mRNA abundance of *NF-κB2* and p52 protein levels in liver tissue of the mice in liver-specific p52 knockdown mice. (**b**) Blood glucose in mice injected with 2 mg/kg glucagon at indicted times. *AUC* is indicated on the right (n=8). (**c**) Relative mRNA abundance of gluconeogenesis genes in liver tissue of mice 1 h after 2 mg/kg glucagon injection. (**d**) Hepatic p52 protein levels in liver-specific p52 overexpression mice. Liver tissues were collected from the AAV8-p52 mice stimulated by glucagon (2 mg/kg for 1 h). NS: Normal Saline; AAV: adeno-associated virus; *AUC*: area under curve; *G6pase*: *glucose-6-phosphatase*; *PEPCK*: *phosphoenolpyruvate carboxykinase*; *PGC-1α*: *peroxisome proliferator-activated receptor gamma coactivator-1 alpha*. Bars represent mean ± SEM values. Statistical differences were determined by one-way ANOVA. *: $p < 0.05$ *vs.* the control group, **: $p < 0.01$ *vs.* the control group. Source data are provided as a Source Data file.

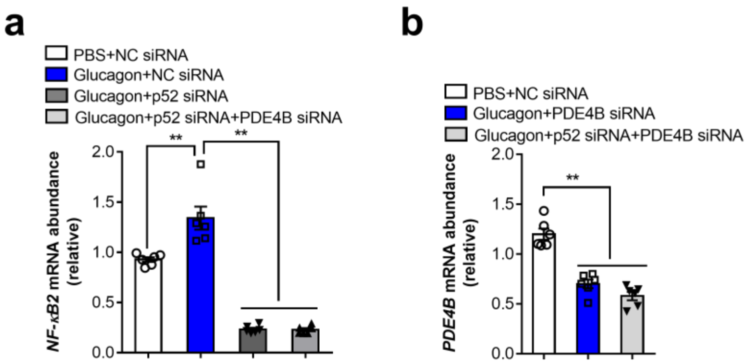

Supplementary Fig. 4 siRNA successfully inhibited *NF-κB2* and *PDE4B* gene transcription. Relative mRNA abundance of *NF-κB2* (**a**) and *PDE4B* (**b**) in siRNA transfection primary hepatocytes. PBS: phosphate buffer solution; PDE: phosphodiesterase. All values are denoted as means ± SEM. Statistical differences were determined by one-way ANOVA. **: $p < 0.01$ *vs*. the control group. Source data are provided as a Source Data file.

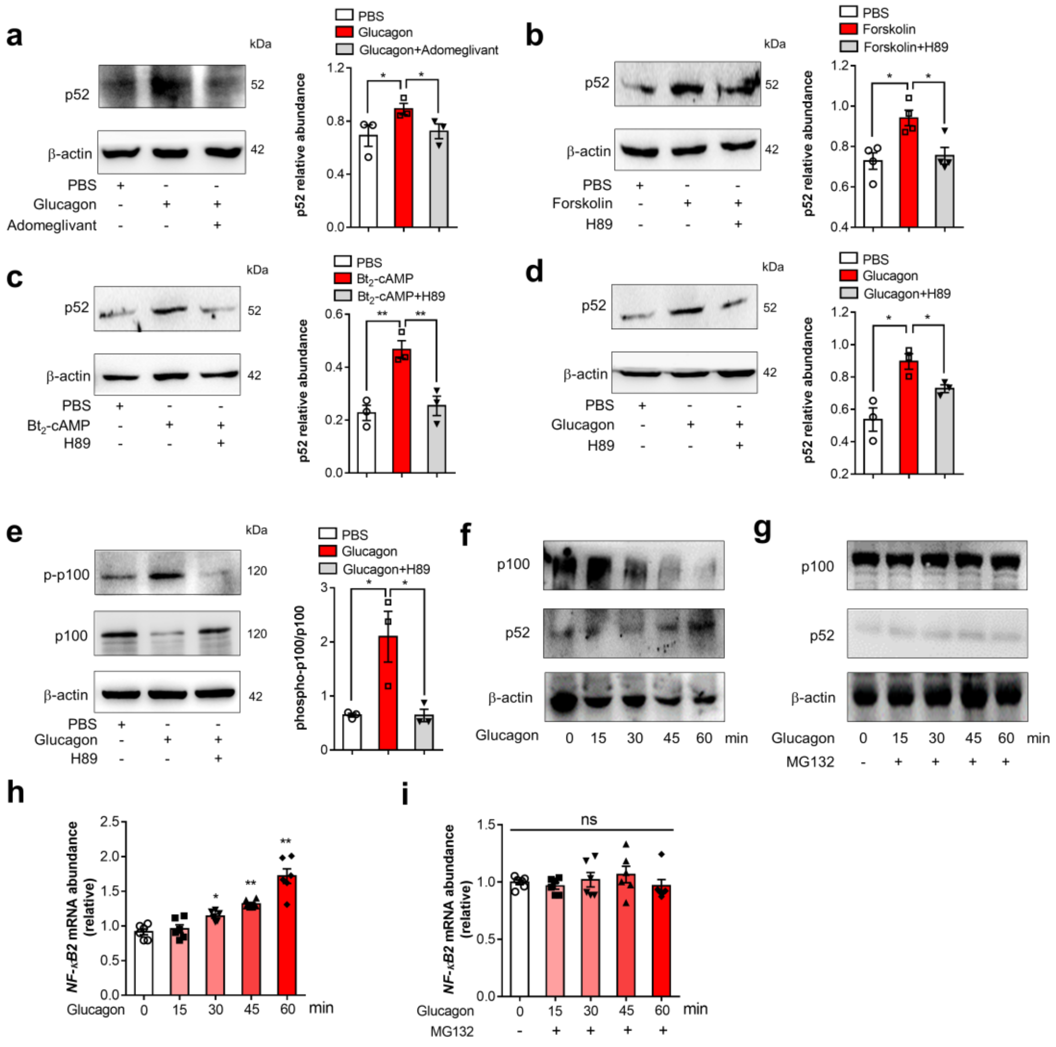

Supplementary Fig. 5 Glucagon activates p52 through the cAMP/PKA pathway. (**a**) Relative protein expression in primary hepatocytes stimulated with the glucagon receptor inhibiter, adomeglivant (10 μM, pre-treated for 2 h) and glucagon (100 nM, 1 h). β-actin levels served as loading controls. (**b**, **c**) Western blotting analysis of p52 in primary hepatocytes exposed to forskolin (10 μM, 1 h) (**b**) and Bt_2-cAMP (100 μM, 1 h) (**c**) pro-treated with H89 (20 μM, 2 h) or DMSO. (**d**, **e**) Immunoblots of p52 (**d**) and p-p100 (**e**) levels in primary hepatocytes when exposed to glucagon (100 nM, 1 h) pre-treated with or without H89 (20 μM, 2 h). (**f**, **g**) p100 and p52 protein levels in primary hepatocytes stimulated by glucagon at indicted times without (**f**) or with MG132 (**g**). MG132 was added into culture medium 1 h before glucagon stimulation. (**h**, **i**) Relative mRNA abundance of *NF-κB2* stimulated by glucagon for different duration without (**h**) or with MG132 (**i**). PBS: phosphate buffer solution. All values are denoted as means ± SEM. Statistical differences were determined by one-way ANOVA. *: $p < 0.05$, **: $p < 0.01$ *vs.* the control group, ns: not statistically significant. Source data are provided as a Source Data file.

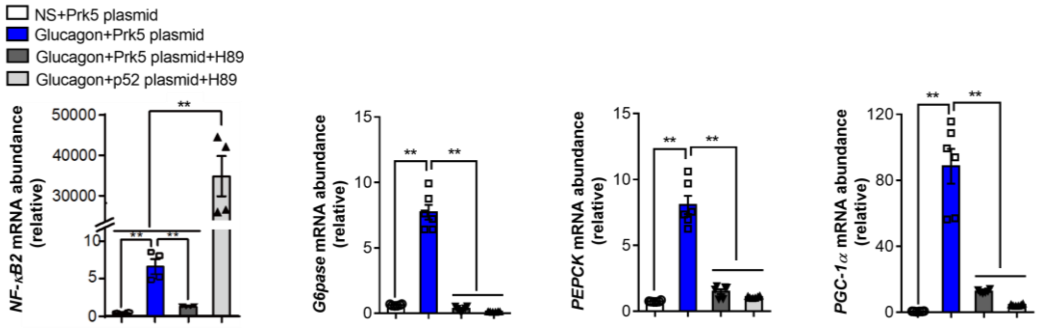

Supplementary Fig. 6 p52 overexpression was not able to restore the gluconeogenesis under H89-treated conditions. Relative mRNA abundance of *NF-κB2*, *G6pase*, *PEPCK* and *PGC-1α* in p52 overexpression HepG2 cells stimulated by glucagon (100 nM, 1 h) when pretreated with or without H89 (20 μM, 2 h). *β-actin* levels were used as a reference. NS: normal saline; *G6pase*: *glucose-6-phosphatase*; *PEPCK*: *phosphoenolpyruvate carboxykinase*; *PGC-1α*: *peroxisome proliferator-activated receptor gamma coactivator-1 alpha*. Bars represent mean ± SEM values. Statistical differences were determined by one-way ANOVA. **: $p < 0.01$ *vs*. the control group. Source data are provided as a Source Data file.

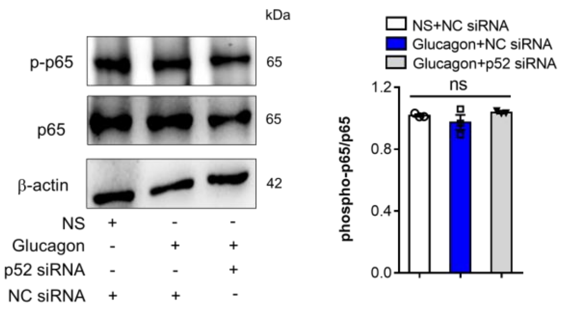

Supplementary Fig. 7 Glucagon has no effect on canonical NF-κB signaling. Phosphorylation levels of p65 in mice liver stimulated by glucagon (2 mg/kg glucagon for 1 h). NS: normal saline. Bars represent mean ± SEM values. Statistical differences were determined by one-way ANOVA. ns: not statistically significant. Source data are provided as a Source Data file.

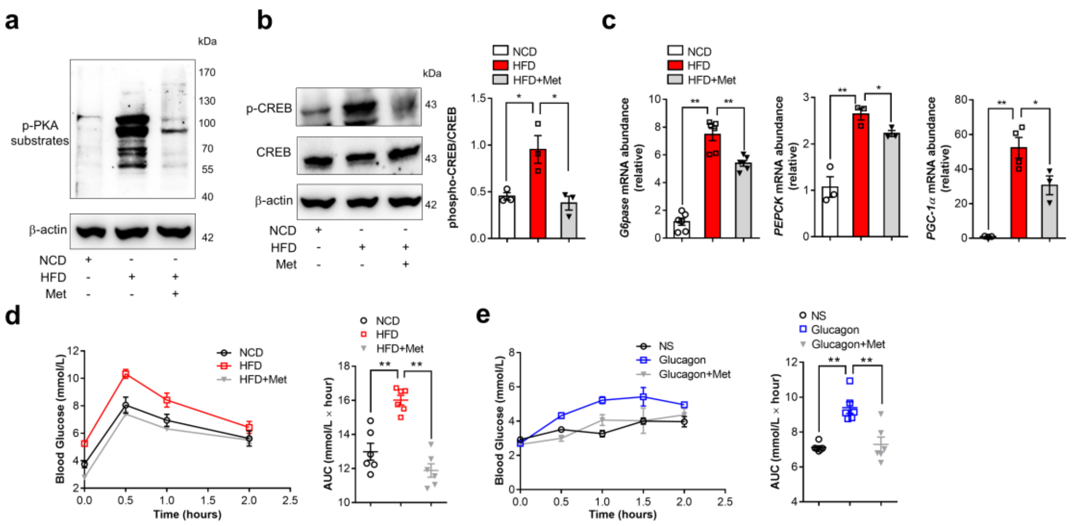

Supplementary Fig. 8 Metformin suppresses excessive activation of gluconeogenesis induced by HFD. Western blotting analysis of phospho-PKA substrates (**a**) and phosphorylation levels of CREB (**b**) in mice liver samples. (**c**) The mRNA levels of *G6pase*, *PEPCK* and *PGC-1α* in mice liver samples. (**d**) Pyruvate tolerance test (2 g/kg body weight) were performed after 7 weeks of feeding. (**e**) The blood glucose levels after glucagon challenge. *AUC* is indicated on the right. NCD: normal chow diet; HFD: high fat diet; Met: metformin; PKA: protein kinase A; CREB: cAMP response element-binding protein; *G6pase*: *glucose-6-phosphatase*; *PEPCK*: *phosphoenolpyruvate carboxykinase*; *PGC-1α*: *peroxisome proliferator-activated receptor gamma coactivator-1 alpha*; *AUC*: area under curve; NS: normal saline. All values are denoted as means ± SEM. Statistical differences were determined by one-way ANOVA. *: $p < 0.05$, **: $p < 0.01$ *vs.* the control group. Source data are provided as a Source Data file.

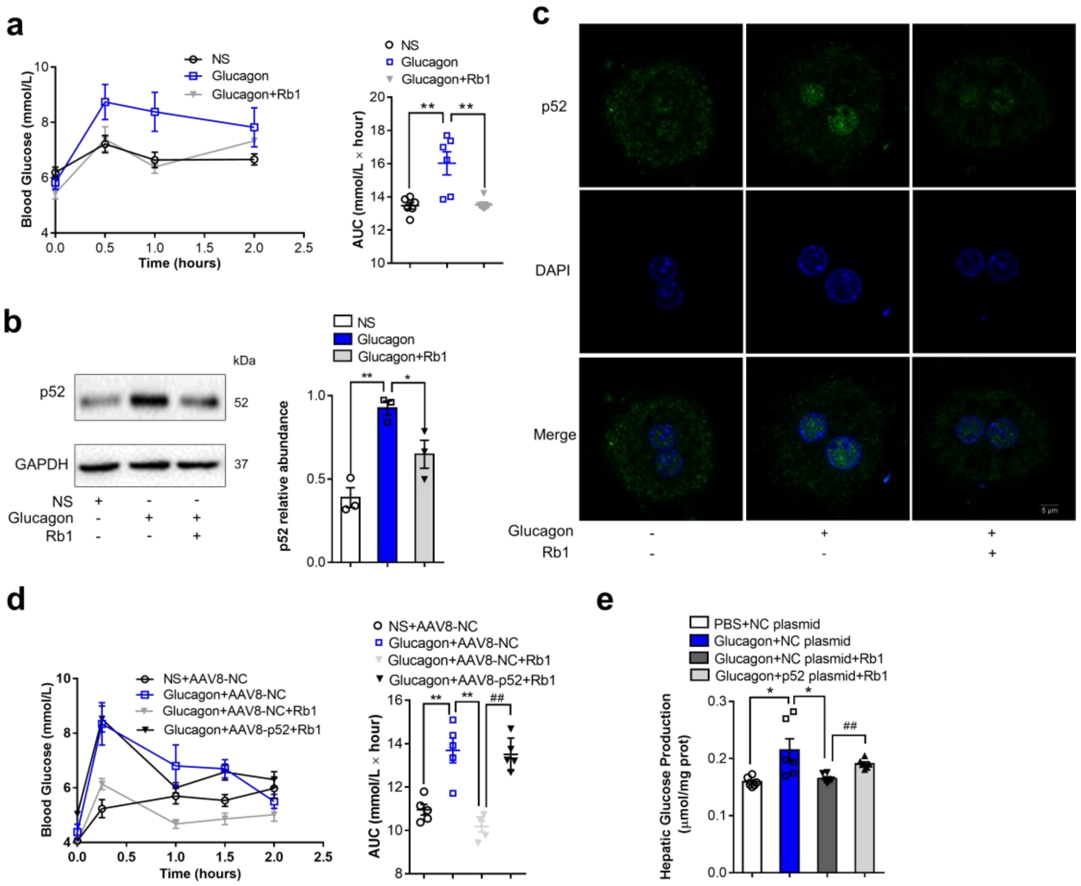

Supplementary Fig. 9 Ginsenoside Rb1 suppresses glucagon-induced hyperglycemia and p52 activation. (**a**) Blood glucose levels in mice subjected to a glucagon challenge. Mice were given ginsenoside Rb1 by gavage (50 mg/kg body weight), and 1 h later glucagon (2 mg/kg) were injected intraperitoneally into mice. *AUC* is indicated on the right (n=6). Bars represent mean ± SEM values. (**b**) Western blotting analysis of p52 in mouse liver samples. (**c**) Representative confocal images of primary hepatocytes exposed to glucagon (100 nM, 1 h) pre-treated with Rb1 (10 μM, for 4 h) or PBS. Scale bar represents 5 μm. (**d**) Blood glucose levels of liver-specific p52 overexpression mice pre-administrated with or without 50 mg/kg Rb1 in glucagon challenge test. *AUC* is indicated on the right (n=5). (**e**) Hepatic glucose production in p52 overexpression primary hepatocytes treated with or without 10 μM Rb1. NS: normal saline; *AUC*: area under curve; DAPI: 4',6-diamidino-2-phenylindole; AAV: adeno-associated virus; PBS: phosphate buffer solution. All values are denoted as means ± SEM. Statistical differences were determined by one-way ANOVA. *: $p < 0.05$, **: $p < 0.01$ *vs.* the control group. $^{\#\#}$: $p < 0.01$ *vs.* AAV8-p52 or p52 plasmid group. Source data are provided as a Source Data file.

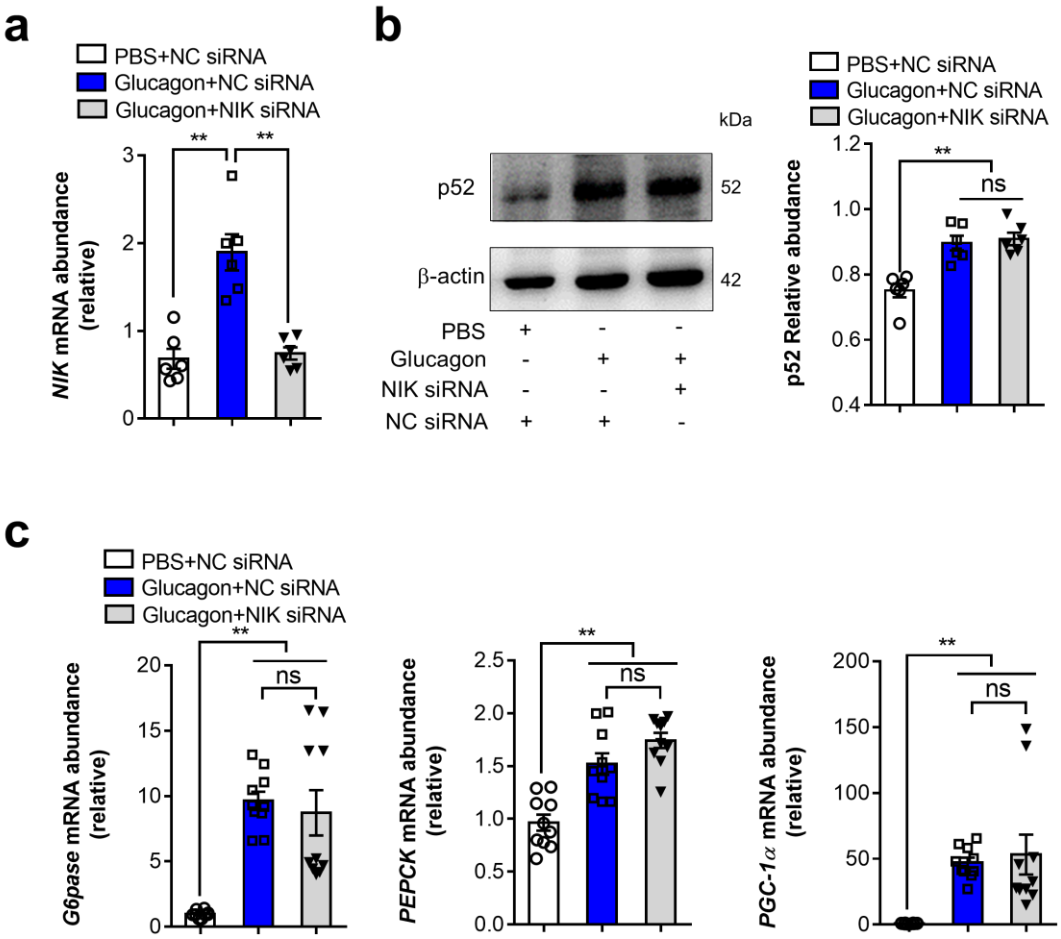

Supplementary Fig. 10 Glucagon activates p52 and gluconeogenesis partly independent on NIK. Relative mRNA abundance of *NIK* (**a**) in glucagon-stimulated primary hepatocytes (100 nM, 1 h) transfected with *NIK* siRNA. Primary hepatocytes transfected with NC siRNA were used as control. (**b**) p52 protein levels in primary hepatocytes stimulated by glucagon (100 nM, 1 h). (**c**) Gluconeogenic genes relative mRNA abundance. PBS: phosphate buffer solution; *NIK*: *NF-κB inducing kinase*; *G6pase*: *glucose-6-phosphatase*; *PEPCK*: *phosphoenolpyruvate carboxykinase*; *PGC-1α*: *peroxisome proliferator-activated receptor gamma coactivator-1 alpha*. All values are denoted as means ± SEM. Statistical differences were determined by one-way ANOVA. **: $p < 0.01$ *vs*. the control group. ns: not statistically significant. Source data are provided as a Source Data file.

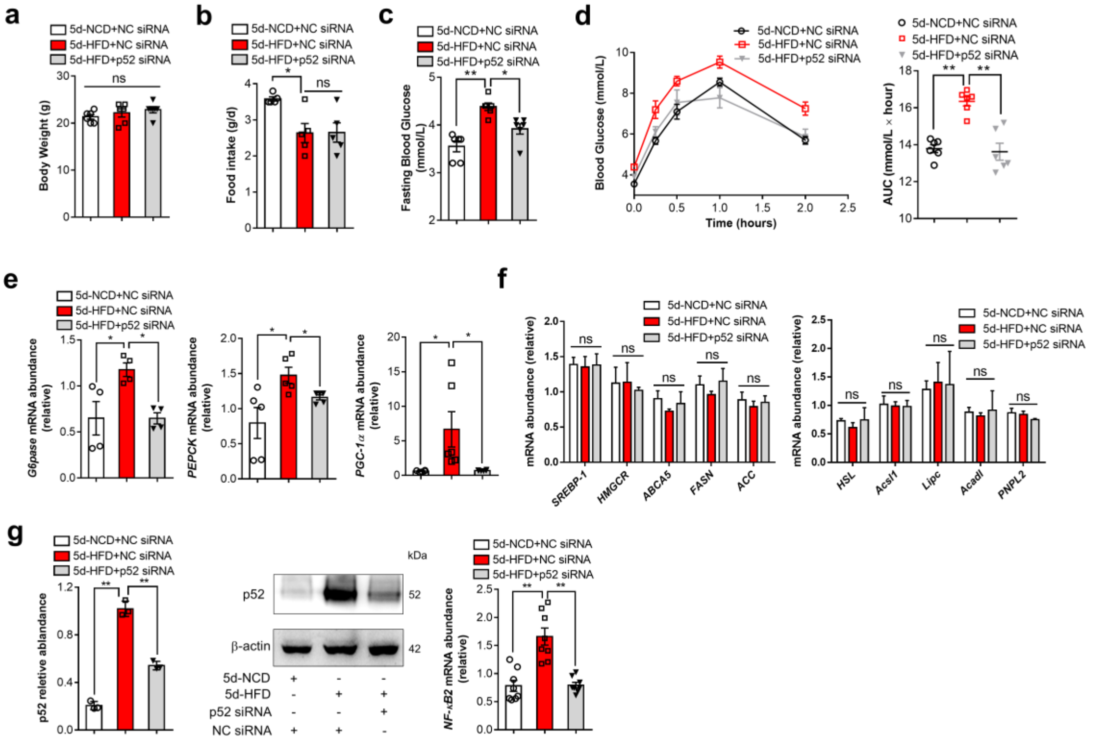

Supplementary Fig. 11 Knockdown of p52 suppresses gluconeogenesis in short-period HFD-fed mice. Body weight (**a**), food intake (**b**) and fasting blood glucose (**c**) in p52 knockdown mice fed with HFD for 5 days. (**d**) Pyruvate tolerance test (2 g/kg body weight) in the mice after overnight fasting. *AUC* is indicated on the right (n=6). (**e**, **f**) Gluconeogenesis (**e**) and lipid metabolism (**f**) related genes mRNA abundance in liver tissue of the mice fasted overnight. (**g**) Relative mRNA abundance of *NF-κB2* and p52 protein levels in liver tissue of mice in (**a**). Liver tissues were collected from the mice after 5 days feeding. NCD: normal chow diet; HFD: high fat diet; *AUC*: area under curve; *G6pase*: *glucose-6-phosphatase*; *PEPCK*: *phosphoenolpyruvate carboxykinase*; *PGC-1α*: *peroxisome proliferator-activated receptor gamma coactivator-1 alpha*; *SREBP-1*: *sterol regulatory element binding proteins-1*; *HMGCR*: *3-hydroxy-3-methylglutaryl-coenzyme A reductase*; *ABCA5*: *ATP binding cassette subfamily A member 5*; *FASN*: *fatty acid synthase*; *ACC*: *acetyl CoA carboxylase*; *HSL*: *hormone-sensitive lipase*; *Acsl1*: *long-chain-fatty-acid-CoA ligase 1*; *Lipc*: *hepatic lipase gene*; *Acadl*: *acyl-CoA dehydrogenase*; *PNPL2*: *recombinant patatin like phospholipase domain containing protein 2*. Bars represent mean ±SEM values. Statistical differences were determined by one-way ANOVA. *: $p < 0.05$ *vs.* the control group, **: $p < 0.01$ *vs.* the control group. ns: not statistically significant. Source data are provided as a Source Data file.

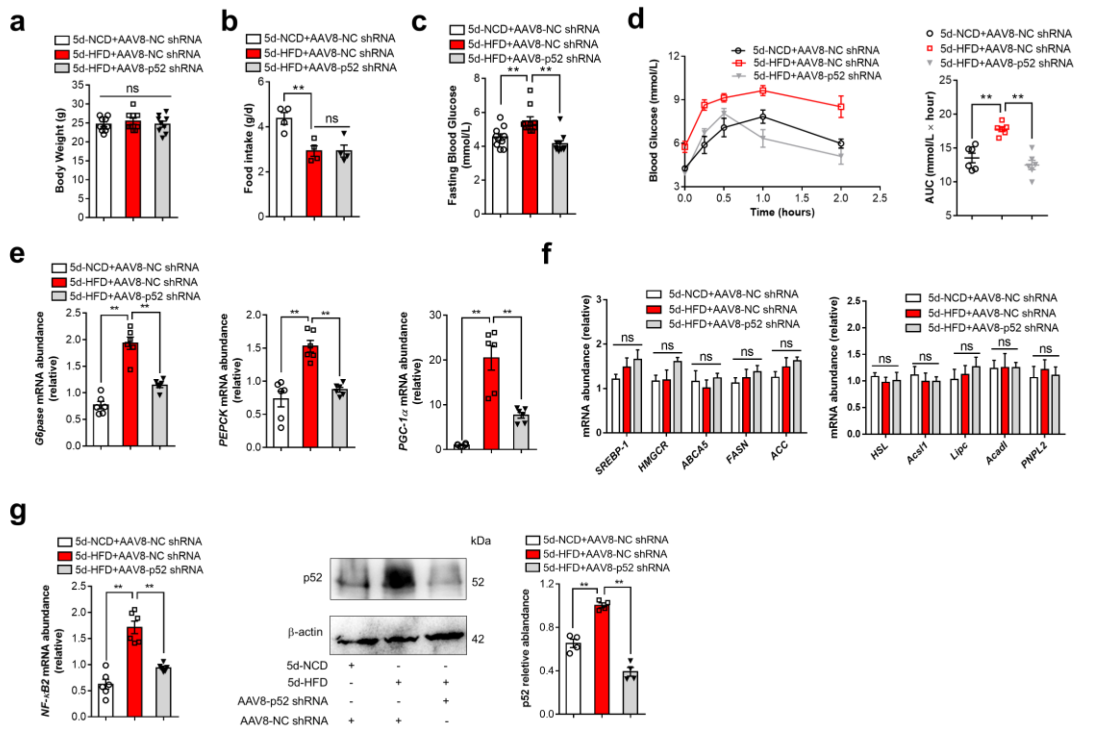

Supplementary Fig. 12 Liver-specific knockdown of p52 suppressed gluconeogenesis in short-period HFD-fed mice. Body weight (**a**), food intake (**b**) and fasting blood glucose (**c**) in p52 liver-specific knockdown mice after HFD-fed for 5 days. AAV8-p52 shRNA was injected 3 weeks before HFD feeding. (**d**) Pyruvate tolerance test (2 g/kg body weight) of mice after short-period HFD feeding. *AUC* is indicated on the right (n=6). Gluconeogenic (**e**) and lipid metabolism (**f**) genes relative mRNA abundance in liver tissue of short-period HFD-fed mice. (**g**) Relative mRNA abundance of *NF-κB2* and p52 protein levels in liver tissue. Liver tissues were collected from the mice after 5 days feeding. NCD: normal chow diet; HFD: high fat diet; *AUC*: area under curve; *G6pase*: *glucose-6-phosphatase*; *PEPCK*: *phosphoenolpyruvate carboxykinase*; *PGC-1α*: *peroxisome proliferator-activated receptor gamma coactivator-1 alpha*; *SREBP-1*: *sterol regulatory element binding proteins-1*; *HMGCR*: *3-hydroxy-3-methylglutaryl-coenzyme A reductase*; *ABCA5*: *ATP binding cassette subfamily A member 5*; *FASN*: *fatty acid synthase*; *ACC*: *acetyl CoA carboxylase*; *HSL*: *hormone-sensitive lipase*; *Acsl1*: *long-chain-fatty-acid-CoA ligase 1*; *Lipc*: *hepatic lipase gene*; *Acadl*: *acyl-CoA dehydrogenase*; *PNPL2*: *recombinant patatin like phospholipase domain containing protein 2*. Bars represent mean ± SEM values. Statistical differences were determined by one-way ANOVA. **: $p < 0.01$ *vs*. the control group. ns: not statistically significant. Source data are provided as a Source Data file.

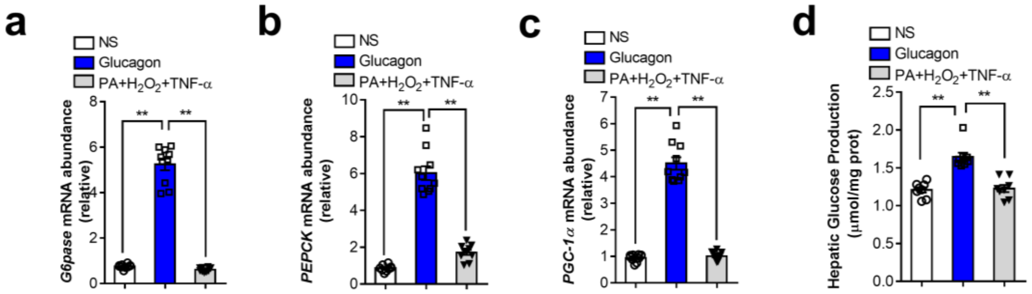

Supplementary Fig. 13 Evaluation of hepatic gluconeogenesis stimulated by glucagon or combined integrated stress. (**a-c**) Relative mRNA abundance of gluconeogenesis-related genes in primary hepatocytes stimulated by glucagon or a combination of TNF-α (10 ng/mL), H_2O_2 (100 nM) and PA (100 μM). (**d**) Hepatic glucose production stimulated by glucagon or a combination of TNF-α (10 ng/mL), H_2O_2 (100 nM) and PA (100 μM). NS: normal saline; PA: palmitic acid; *G6pase*: glucose-6-phosphatase; *PEPCK*: phosphoenolpyruvate carboxykinase; *PGC-1α*: peroxisome proliferator-activated receptor gamma coactivator-1 alpha. All values are denoted as means ± SEM. Statistical differences were determined by one-way ANOVA. **: $p < 0.01$ *vs.* the control group. Source data are provided as a Source Data file.

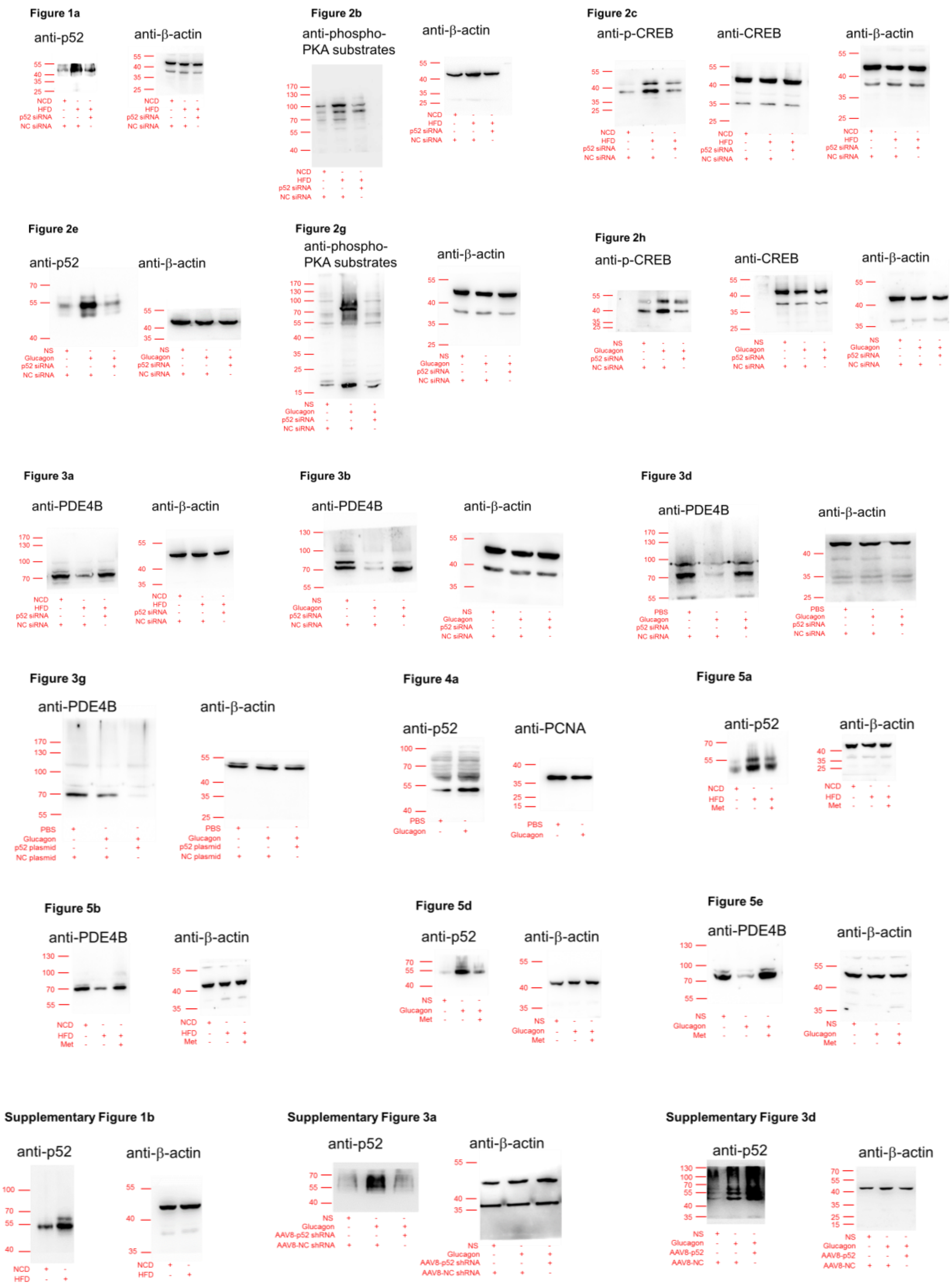

Supplementary Fig. 14 Original source data of the blotting images.

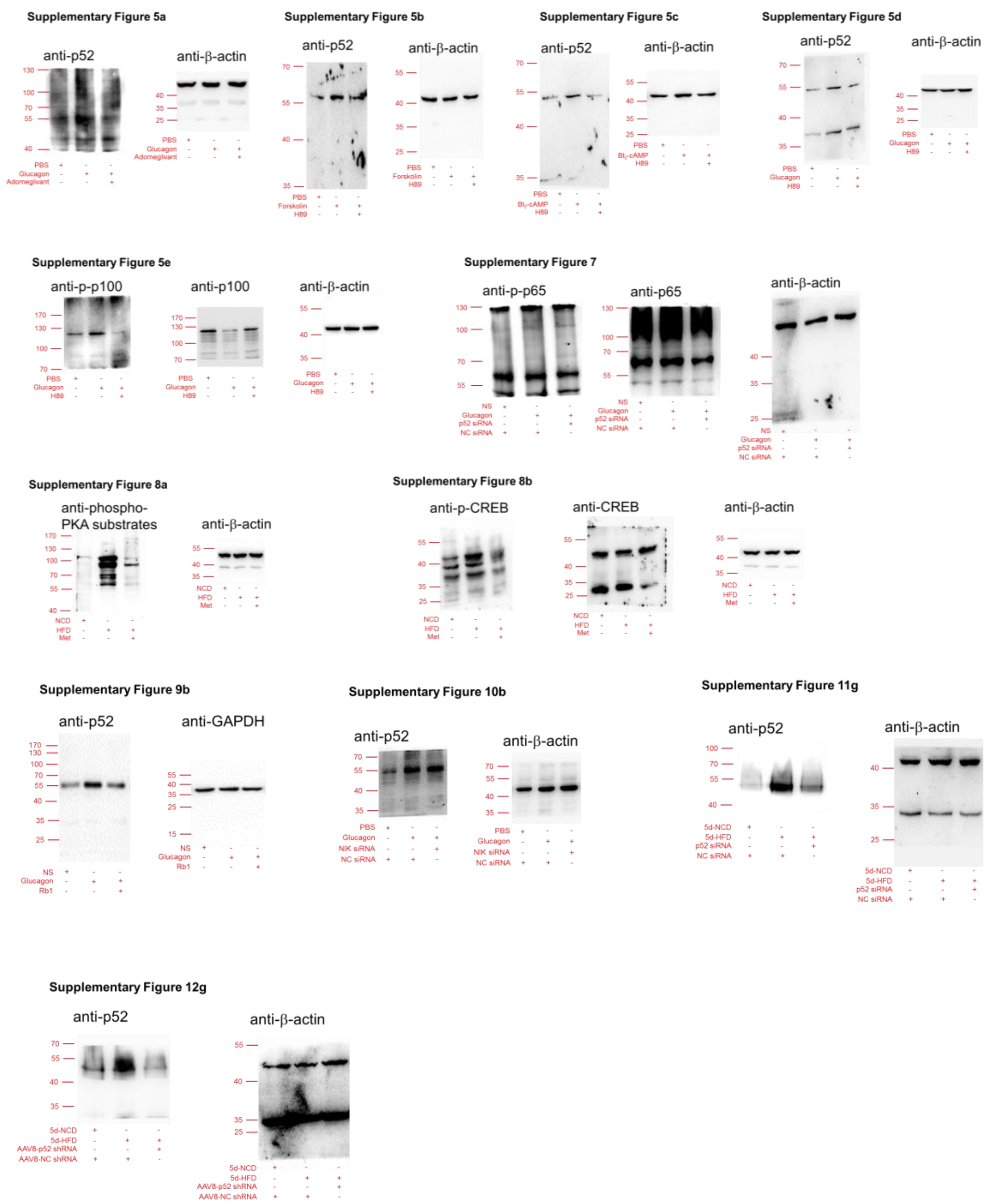

Supplementary Fig. 14 Original source data of the blotting images (continued).

Supplementary Table 1. Primer pairs for qRT-PCR

Gene		Sequence 5’-3’
NF-κB2 (human)	Forward	ATGGAGAGTTGCTACAACCCA
	Reverse	CTGTTCCACGATCACCAGGTA
NF-κB2 (mice)	Forward	TGGCATCCCCGAATATGATGA
	Reverse	TGACAGTAGGATAGGTCTTCCG
NIK (human)	Forward	CCACCTTTTCAGAACGCATTTTC
	Reverse	GTAGCATGGGCCACATTGTTG
NIK (mice)	Forward	TCTCTGGAGGAACAGGAACAA
	Reverse	GCCATTGAGAGACTGGATCTG
G6Pase (mice)	Forward	CGACTCGCTATCTCCAAGTGA
	Reverse	GTTGAACCAGTCTCCGACCA
G6Pase (human)	Forward	CTACTACAGCAACACTTCCGTG
	Reverse	GGTCGGCTTTATCTTTCCCTGA
PEPCK (mice)	Forward	AAGCATTCAACGCCAGGTTC
	Reverse	GGGCGAGTCTGTCAGTTCAAT
PEPCK (human)	Forward	AGTAGAGAGCAAGACGGTGAT
	Reverse	TGCTGAATGGAAGCACATACAT
PGC-1α (mice)	Forward	ATACCGCAAAGAGCACGAGAAG
	Reverse	CTCAAGAGCAGCGAAAGCGTCACAG
PGC-1α (human)	Forward	TCTGAGTCTGTATGGAGTGACAT

	Reverse	CCAAGTCGTTCACATCTAGTTCA
PDE4B (human)	Forward	TGATGCTCAGGACATTCTCG
	Reverse	AGTGGTGGTGAGGGACTTTG
PDE4B (mice)	Forward	GTAGAGGCCAGTTCCCATCA
	Reverse	CCAACACCTAGTGCAGAGCA
PDE3B (human)	Forward	GCTCGCAATATGGTGTCAGAT
	Reverse	TGAGAAAGCACCCATTAAGCTAC
PDE3B (mice)	Forward	TGCCTTCTTCTTCCTCACCTG
	Reverse	GACCACCACTGCCACACC
SREBP-1	Forward	GCAGCCACCATCTAGCCTG
	Reverse	CAGCAGTGAGTCTGCCTTGAT
HMGCR	Forward	AGCTTGCCCGAATTGTATGTG
	Reverse	TCTGTTGTGAACCATGTGACTTC
ABCA5	Forward	GATGTGGGAGTTTGGAGACAG
	Reverse	GCTGAATTTGTCCATAGGGCT
FASN	Forward	GGAGGTGGTGATAGCCGGTAT
	Reverse	TGGGTAATCCATAGAGCCCAG
ACC	Forward	GATGAACCATCTCCGTTGGC
	Reverse	ACCCAATTATGAATCGGGAGTG
HSL	Forward	TCCCTCAGTATCTAGGCCAGA
	Reverse	GGCTCATTTGGGAGACTTTGTTT
Acsl1	Forward	TGCCAGAGCTGATTGACATTC

	Reverse	GGCATACCAGAAGGTGGTGAG
Lipc	Forward	ATGGGAAATCCCCTCCAAATCT
	Reverse	GTGCTGAGGTCTGAGACGA
Acadl	Forward	TCTTTTCCTCGGAGCATGACA
	Reverse	ACCTCTCTACTCACTTCTCCAG
PNPL2	Forward	ACCACCCTTTCCAACATGCTA
	Reverse	GGCAGAGTATAGGGCACCA
β-actin	Forward	AGTGTGACGTTGACATCCGTA
	Reverse	GCCAGAGCAGTAATCTCCTTCT

Supplementary Table 2. Primary antibodies for Western blotting and ChIP

Antibody	Cat No.	Company	Dilutions	Source
NF-κB2 (p52)	sc-7386	Santa Cruz	1:500	Mouse
NF-κB2 (p52) for ChIP	sc-7386X	Santa Cruz	1:100	Mouse
phospho-NF-κB p65 (Ser536)	3033S	CST	1:1000	Rabbit
NF-κB (p65)	4764S	CST	1:1000	Rabbit
PDE4B	sc-25812	Santa Cruz	1:500	Rabbit
phospho-PKA substrates	9621S	CST	1:1000	Rabbit
phospho-CREB (Ser133)	9198S	CST	1:1000	Rabbit
CREB	9197S	CST	1:1000	Rabbit
β-actin	4970S	CST	1:1000	Rabbit
β-actin	bs-00612R	Bioss	1:500	Rabbit
GAPDH	5174S	CST	1:1000	Rabbit
PCNA	AF1363	Beyotime	1:500	Rabbit
