# Supplementary material for: Inactivation of NF-κB2 (p52) restrains hepatic glucagon response via preserving PDE4B induction
Source: Nat Commun. 2019 Sep 20;10:4303. doi: 10.1038/s41467-019-12351-x (PMC6754499; doi:10.1038/s41467-019-12351-x)
Supplement: Supplementary file 3 — Reporting Summary [file 41467_2019_12351_MOESM3_ESM.pdf]

nature research

Corresponding author(s): Lian-Wen Qi, Qun Liu, Ping Li

Last updated by author(s): Jun 26, 2019

Reporting Summary

Nature Research wishes to improve the reproducibility of the work that we publish. This form provides structure for consistency and transparency in reporting. For further information on Nature Research policies, see Authors & Referees and the Editorial Policy Checklist.

Statistics

For all statistical analyses, confirm that the following items are present in the figure legend, table legend, main text, or Methods section.

n/a	Confirmed	
☐	☒	The exact sample size (*n*) for each experimental group/condition, given as a discrete number and unit of measurement
☐	☒	A statement on whether measurements were taken from distinct samples or whether the same sample was measured repeatedly
☐	☒	The statistical test(s) used AND whether they are one- or two-sided<br>*Only common tests should be described solely by name; describe more complex techniques in the Methods section.*
☒	☐	A description of all covariates tested
☒	☐	A description of any assumptions or corrections, such as tests of normality and adjustment for multiple comparisons
☐	☒	A full description of the statistical parameters including central tendency (e.g. means) or other basic estimates (e.g. regression coefficient) AND variation (e.g. standard deviation) or associated estimates of uncertainty (e.g. confidence intervals)
☒	☐	For null hypothesis testing, the test statistic (e.g. *F*, *t*, *r*) with confidence intervals, effect sizes, degrees of freedom and *P* value noted<br>*Give P values as exact values whenever suitable.*
☒	☐	For Bayesian analysis, information on the choice of priors and Markov chain Monte Carlo settings
☒	☐	For hierarchical and complex designs, identification of the appropriate level for tests and full reporting of outcomes
☐	☒	Estimates of effect sizes (e.g. Cohen's *d*, Pearson's *r*), indicating how they were calculated

Our web collection on statistics for biologists contains articles on many of the points above.

Software and code

Policy information about availability of computer code

Data collection	No software was used.
Data analysis	GraphPad Prism 7 were used for statistical analysis.

For manuscripts utilizing custom algorithms or software that are central to the research but not yet described in published literature, software must be made available to editors/reviewers. We strongly encourage code deposition in a community repository (e.g. GitHub). See the Nature Research guidelines for submitting code & software for further information.

Data

Policy information about availability of data

All manuscripts must include a data availability statement. This statement should provide the following information, where applicable:

- Accession codes, unique identifiers, or web links for publicly available datasets
- A list of figures that have associated raw data
- A description of any restrictions on data availability

The authors declare that the data supporting the findings of this study are available within the article and its Supplementary Information Files, or are available from the corresponding authors on resonable request.

Field-specific reporting

Please select the one below that is the best fit for your research. If you are not sure, read the appropriate sections before making your selection.

☒ Life sciences ☐ Behavioural & social sciences ☐ Ecological, evolutionary & environmental sciences

Life sciences study design

All studies must disclose on these points even when the disclosure is negative.

Sample size	Sample size was chosen depending on the experimental approaches used based on our experience and estimation. No statistical method was used to predetermine sample size.
Data exclusions	No data was excluded.
Replication	The shown results could successfully and reliably be replicated and reproduced.
Randomization	All the cells and mice were randomly allocated into different groups.
Blinding	No blinding was done.

Reporting for specific materials, systems and methods

We require information from authors about some types of materials, experimental systems and methods used in many studies. Here, indicate whether each material, system or method listed is relevant to your study. If you are not sure if a list item applies to your research, read the appropriate section before selecting a response.

Materials & experimental systems

n/a	Involved in the study	
☐	☒	Antibodies
☐	☒	Eukaryotic cell lines
☒	☐	Palaeontology
☐	☒	Animals and other organisms
☒	☐	Human research participants
☒	☐	Clinical data

Methods

n/a	Involved in the study	
☒	☐	ChIP-seq
☒	☐	Flow cytometry
☒	☐	MRI-based neuroimaging

Antibodies

Antibodies used	NF-κB2 (p52), Santa Cruz, cat#sc-7386; NF-κB2 (p52) for ChIP, Santa Cruz, cat#sc-7386X;<br>phospho-NF-κB p65 (Ser536), Cell Signaling Technology, cat#3033S; NF-κB (p65), Cell Signaling Technology, cat#4764S;<br>PDE4B, Santa Cruz, cat#sc-25812; phospho-PKA substrates, Cell Signaling Technology, cat#9621S;<br>phospho-CREB (Ser133), Cell Signaling Technology, cat#9198S; CREB, Cell Signaling Technology, cat#9197S;<br>p-NF-κB2 P100 (s866/870), Cell Signaling Technology, cat#4810P; NF-κB2 P100, Cell Signaling Technology, cat#4882T;<br>β-actin, Cell Signaling Technology, cat#4970S; β-actin, Bioss, cat#bs-0061R; GAPDH, Cell Signaling Technology, cat#5174S;<br>PCNA, Beyotime, cat#AF1363.
Validation	All antibodies used in the study are commercially available and validation statement is given on manufacturer's website.

Eukaryotic cell lines

Policy information about cell lines

Cell line source(s)	HepG2 cell line was obtained from the American Type Culture Collection and 293T cell line was obtained from Stem Cell Bank, Chinese Academy of Sciences.
Authentication	The cell lines were purchased from the authorized source, and none of them were authenticated thereafter.
Mycoplasma contamination	293T was negative for mycoplasma. HepG2 cell line used tested negative for mycoplasma contamination prior to this study.
Commonly misidentified lines<br>(See ICLAC register)	No commonly misidentified lines were used in this study.

Animals and other organisms

Policy information about studies involving animals; ARRIVE guidelines recommended for reporting animal research

Laboratory animals	6-8-week-old male C57BL/6J mice were used in the study.

Wild animals	The study did not involve wild animals.
Field-collected samples	The study did not involve samples collected from the field.
Ethics oversight	The procedures for experiments and animal care were approved by the Institutional Animal Care and Use Committee of China Pharmaceutical University (Nanjing, China).

Note that full information on the approval of the study protocol must also be provided in the manuscript.
